# Experience of intimate partner violence among young pregnant women in urban slums of Kathmandu Valley, Nepal: a qualitative study

**DOI:** 10.1186/s12905-016-0293-7

**Published:** 2016-03-05

**Authors:** Keshab Deuba, Anustha Mainali, Helle M. Alvesson, Deepak K. Karki

**Affiliations:** Public Health and Environment Research Center, Kathmandu, Nepal; Department of Public Health Sciences, Karolinska Institutet, Stockholm, Sweden; Department of Public Health, Nobel College, Pokhara University, Kathmandu, Nepal; Nepal Health Economics Association, Kathmandu, Nepal

**Keywords:** Intimate partner violence, Young pregnant women, Urban slums, Qualitative interviews, Nepal

## Abstract

**Background:**

Intimate partner violence (IPV) is an urgent public health priority. It is a neglected issue in women’s health, especially in urban slums in Nepal and globally. This study was designed to better understand the IPV experienced by young pregnant women in urban slums of the Kathmandu Valley, as well as to identify their coping strategies, care and support seeking behaviours. Womens’ views on ways to prevent IPV were also addressed.

**Methods:**

20 young pregnant women from 13 urban slums in the Kathmandu valley were recruited purposively for this qualitative study, based on pre-defined criteria. In-depth interviews were conducted and transcribed, with qualitative content analysis used to analyse the transcripts.

**Results:**

14 respondents were survivors of violence in urban slums. Their intimate partner(s) committed most of the violent acts. These young pregnant women were more likely to experience different forms of violence (psychological, physical and sexual) if they refused to have sex, gave birth to a girl, or if their husband had alcohol use disorder. The identification of foetal gender also increased the experience of physical violence at the prenatal stage. Interference from in-laws prevented further escalation of physical abuse. The most common coping strategy adopted to avoid violence among these women was to tolerate and accept the husbands’ abuse because of economic dependence. Violence survivors sought informal support from their close family members. Women suggested multiple short and long term actions to reduce intimate partner violence such as female education, economic independence of young women, banning identification of foetal gender during pregnancy and establishing separate institutions within their community to handle violence against young pregnant women.

**Conclusions:**

Diversity in the design and implementation of culturally and socially acceptable interventions might be effective in addressing violence against young pregnant women in humanitarian settings such as urban slums. These include, but are not limited to, treatment of alcohol use disorder, raising men’s awareness about pregnancy, addressing young women’s economic vulnerability, emphasising the role of health care professionals in preventing adverse consequences resulting from gender selection technologies and working with family members of violence survivors.

**Electronic supplementary material:**

The online version of this article (doi:10.1186/s12905-016-0293-7) contains supplementary material, which is available to authorized users.

## Background

Worldwide, millions of women and young girls suffer from violence in many forms— intimate physical and sexual partner violence, child and forced marriage, sex trafficking and rape—which are global public health problems of epidemic proportions [1]. Intimate partner violence (IPV) includes physical, emotional, sexual, psychological, or financial abuse between intimate partners [2]. Gender inequality, the main cause of violence against women and young girls, is often fuelled by discriminatory social norms and structures [3]. A 2013 global review of data from 81 countries found that 30 % of every partnered women (aged 15–69) has experienced physical or sexual IPV in their lifetime, and that the South Asian region has one of the highest levels of this violence in the world (42 %) [4]. A systematic review of data from 66 countries found that more than 35 % of female homicide is perpetrated by an intimate partner [5]. In the 2011 Nepal Demograhphic Health Survey, it was reported that one in three women aged 15–49 years has experienced physical abuse [6]. One study conducted among young married women (15–24 years) in rural areas of Nepal found an even higher prevalence (54 %) of physical and sexual violence in their lifetime [7].

Evidence suggests that women with poor economic status, experiencing housing instability and living in urban slums are at high risk of IPV [8]. The IPV prevalence among women living in urban slum ranges from 27 % in Thailand [8] to 62 % in India [9]. Poor economic status reinforces the underlying gender-based power disparities [10]. The association between poor economic status and IPV is mediated through stress and economically disadvantaged men who also lack the resources to cope with stress [11].

IPV during pregnancy places women in extreme situations of vulnerability. Data from 19 countries showed that the IPV prevalence during pregnancy ranged from 3 % in the Philippines to 14 % in Uganda among ever-pregnant and partnered woman [12]. A study conducted among 950 urban pregnant women at an urban hospital in Kathmandu revealed that 33 % had suffered different types of violence (psychological, physical and sexual abuse) [13]. IPV during pregnancy can have serious physical and mental health consequences such as substance abuse, preterm delivery, foetal distress, antepartum haemorrhage, pre-eclampsia, low birth weight, postnatal depression and risk of death of the mother and foetus [14, 15]. IPV can be initiated during pregnancy but is often a continuation of previous IPV [16]. Women in abusive relationships may believe that pregnancy will protect them from violence and they might expect that their partner will be more sympathetic towards them [17]. However, pregnancy may give rise to insecurities in jealous men who doubt their paternity, and their partner’s fidelity [17]. Early marriage is another risk factor of IPV in pregnancy. Globally more than 1.5 million girls are married before the age of 15 years [3]. In the 2011 Nepal Demographic Health Survey it was found that 29 % of adolescent girls (15–19 years) and 77 % of young women (20–24 years) were married [6].

Early marriage puts girls and women at risk of psychological violence including emotional pressure from their husband and in-laws [10, 18]. Young women between the ages of 15–24 years are more vulnerable to psychosocial challenges compared to other women of a reproductive age group due to several and partly overlapping reasons. These include developmental immaturity, low self-esteem, poor negotiation skills and limited financial resources [19]. In a multi-country study, the lifetime prevalence of IPV among adolescents and young women (15–24 years) ranged from 19 % in Serbia and Montenegro to 66 % in Peru and Ethiopia [20].

Previous studies identified social, economic and religious reasons as well as a preference to give birth to boys as factors contributing towards the high IPV prevalence. Young women in Nepal lack decision-making power in matters related to reproductive health including contraceptive use, sexuality and family size [21]. Male dominance is a deeply rooted cultural norm in Nepali society [13].

One contributing factor to IPV is giving birth to girls. The determinants of son preference, especially in Nepal and India, are rooted in both economic and religious reasons [22–24]. A son is considered a protector from financial hardship when parents gets older (‘*Budes kaal ko sahara*’*-* economic support in old age - while a daughter is considered ‘*Paraya Sampatti*’- someone else’s wealth - because they move to their husband’s house after marriage). A daughter is considered an economic burden due to dowry practices where the bride’s family transfers wealth in cash or in-kind to the bridegroom’s family [24]. Considering a girl as ‘someone else’s wealth’ is a constraint in girls’ access to education [25].

Religious beliefs, especially those belonging to Hindu, also play an important role for son preference [22]. The majority of the Nepalese population (>80  %) are practicing Hindus, wherein the role of a son is critical in the family to act as a saviour. The son is considered a saviour because he is the authorised person in the family who can perform important ritual activities, such as *‘Daag Batti” (*whereby a person’s soul reaches heaven only when the son lights the funeral pyre) and *‘Pinda Daan”* (when the son collects the ashes of the deceased parents and washes it in the Ganges to secure soul salvation). These religious beliefs contribute to the preference of male children.

The past decade has seen advances in understanding the epidemiology of different forms of violence against women, and some interventions to prevent this [26]. However, the 2014 series published in the Lancet on violence against women and girls pointed out that such evidence was highly skewed towards high income countries [27]. Different interventions are found effective in improving physical and mental health outcomes of survivors of violence but ineffective in preventing recidivism/re-victimisation [27]. Studies from low income countries have shown promising results for different forms of IPV by involving multiple stakeholders with varied approaches [27]. The Lancet series also highlighted that little is known about how violence affects groups that are often not captured in population based surveys [26] such as women living in urban slums.

Although epidemiological research into IPV increases, and to some extent also qualitative research, there is limited research exploring the perspectives of pregnant women living in urban slums experiencing IPV [28]. Understanding the experience of IPV among these women is crucial to design evidence based IPV prevention strategies and programmes. As such the study aims were to understand pregnant women’s perceptions and experiences of IPV, to identify coping and support strategies and to ask women about the opportunities of reducing IPV in urban slums.

## Methods

### Study setting and sampling procedures

Nepal is the country with the fourth highest proportion of the population living in urban slums. One of the primary features of urban growth in the Kathmandu valley (Kathmandu, Lalipur and Bhaktapur districts) is the proliferation of urban slum settlements throughout the city. According to a 2008 report, there are a total of 45 urban slums in the Kathmandu valley, in which 13,243 people reside in 2,844 households [29]. In this study, 13 of the 45 urban slums were chosen randomly through a basic lottery method (each slum name was assigned a unique number; the 45 numbers were thoroughly mixed and a research member randomly picked 13 numbers from the pool). Thereafter each slum was visited and a meeting was held with community leaders, police officers and local clubs such as Rotary club, Lions Club and Youth Health Club with the purpose of providing basic information about the study. The study objective was described in culturally adjusted terms (to elicit the experience of family care and support by young pregnant women of urban slums of Kathmandu Nepal) and avoided sensitive concepts such as IPV.

Lists of young pregnant women in the study area were made by members of the research team. Eligible study participants were approached for in-depth interview by two interviewers (one of whom is second author). In order to achieve rich data it was estimated that around 20 interviews were needed [30]. Initially a total of 25 participants were approached for an interview. Three women declined due to lack of time or willingness to participate and two women refused to participate in the study without any reasons. Consequently, 20 women agreed to participate.

### In-depth interviews

Individual in-depth interviews were conducted from August to December 2013 by two female public health students (23 and 24 years old). They were trained in conducting qualitative interviews and were used to spending time in urban slums from previous work. To maintain confidentiality, interviews took place in a private room (in the respondent’s own bedroom) or in a secluded area in her home as chosen by the participant. Interviews were set up at a time convenient for the women which often was during the daytime. Interviews lasted 45–70 min, were conducted in Nepali and were tape recorded.

An open-ended semi-structured interview guide (Additional file 1) was developed; informed by literature on IPV. The guide contained questions related to different forms (physical, psychological and sexual) of IPV. Two pilot interviews were conducted with pregnant women at urban slums in the Kathmandu valley to ensure comprehensibility and content validity. Minor adjustments to the context were subsequently made.

We recorded socio-demographic information, such as age, ethnicity, caste, education, type of family and age at marriage before the interview started. Since demographic characteristics of the respondents’ spouses are highly relevant to experiences of different forms of IPV, the interviews included questions about the husband’s age, education and occupation.

### Data management and analysis

The data was analysed using a qualitative content analysis approach [31]. Initially the interviews were transcribed in Nepali and then translated into English. Two co-authors (AM and DKK) did the transcription and translation. After reviewing the transcripts, categories and subcategories were developed for organising and analysing subsequent interviews. Data analysis was an iterative process, whereby categories and subcategories were continuously generated, revised and re-examined by co-authors (AS, KD, HMA and DKK). During this process alternative interpretations were discussed among the authors. A continuous comparison and contrasting of results was done to improve trustworthiness and to avoid researcher predispositions with the support from co-author (HMA).

### Ethical considerations

The study protocol was reviewed and approved by the Nepal Health Research Council (Reference number-1023). As stated in the WHO guidelines on ‘Ethical and Safety Recommendations for Research on Domestic Violence against Women’ [32], ethical considerations are particularly important in studies on IPV. To ensure the safety of the respondents and research team, we established good working relations with local formal and informal leaders. To minimise under-reporting of experiences of violence, female interviewers conducted the interviews in the natural environment of the participants. To protect confidentiality and data safety, personal identifiers were not collected. To reduce any possible distress caused by participation in the study, trained counsellors were available if needed. None of the participants sought counselling with the study counsellors.

Before initiating the interviews each participant was asked for verbal consent to participate. Participants were informed of their full right to skip any questions and terminate their participation at any stage without any given reason. No cash incentives or gifts were provided. Similar procedures were followed towards participants who were under 18 years old according to recommendations of ‘National Ethical Guidelines for Health Research in Nepal and Standard Operating Procedures’ from the Research Council [33]. NHRC grants the waiver of parental permission for minors’ participation in this research because of the sensitive nature of the research and potential conflict of interests between parents and participants under 18. The parents’ or legal guardian (in our case in-laws or husband) may be reluctant to permit minor to participate in this research which could ultimately hamper the feasibility and validity of the research.

## Results

We will first present women’s perceptions and experiences of the different types of IPV during prior and current (at time of study) pregnancies. Participants had experienced multiple and severe episodes of IPV and each type is therefore voiced by the women who had experienced the specific types. These experiences set the stage for women’s coping strategies and suggestions on ending IPV in urban slums. Socio-demographic characteristics of the 20 participants are shown in Table 1, while Table 2 contains a socio-demographic description of their spouses.

**Table 1:** Sociodemographic characteristics of 20 interviewed young pregnant women

Characteristics	Response (n)
Age (years)	
15-19	6
20-24	14
Caste^a^	
Upper caste (*Brahmin/Kshatriya)*	2
Marginalized caste	
Tharu and Tamang	13
Newar	2
Lower caste-Sarki	3
Religion	
Hindu	11
Buddhist	3
Christian	4
Not disclosed	2
Type of marriage^b^	
Love/Inter-caste)	13
Arranged	7
Type of family^c^	
Nuclear	12
Joint	8
Level of education	
Illiterate	5
Primary (1–5 grades)	2
Secondary (6–10 grades)	9
≥School leaving certificate (≥10 grades)	4
Age of marriage (years)	
<18 years	7
≥18 years	13
Occupation	
Housewives	18
Business	2
Family income per month	
NPR <10,000	8
NPR 10,000–20,000	8
NPR 20,000–30,000	4

^a^According to general code, Nepali people are divided into four hierarchies: *Tagadhari* (castes wearing sacred thread) considered as upper caste; *Matawali* (Alcohol-drinking castes) considered as marginalized caste*; Pani nachalne chhoi chitto halnunaparne* (Impure but touchable castes) and *Pani nachalne chhoi chitto halnu parne* (untouchable castes) were considered as lower castes. Details about reasons behind hierarchical positions are described elsewhere [51]. ^b^Love/inter-caste marriage- In love marriage couple decide to get married with or without parents approval and if couples belongs to different castes and get married with or without parents approval is considered as inter-caste marriage. In the arranged marriage the parents of the bride and bridegroom agrees on the marriage with or without the approval of bride and bridegroom. ^c^A couple and their children living together is a nuclear family and joint family is an extended form of nuclear family which includes grandparents and other relatives. In Nepal family members related to paternal lineage lived together in one house as a joint family

*NPR*: Nepalese rupee (1 USD = 107 NPR)

**Table 2:** Characterstics of respondents’ husbands

Characteristics	Response (n)
Age of husbands (years)	
15–19	2
20–24	12
25–29	6
Level of education	
Illiterate	4
Primary (1–5 grades)	2
Secondary (6–10 grades)	8
≥School leaving certificate (≥10 grades)	6
Occupation	
Unemployed	2
Business	6
Labourer	8
Service holder	4

### All types of violence are experienced by the pregnant women in the study

#### Physical violence during pregnancy

Interviews revealed that seven of the 20 women were beaten during pregnancy by their intimate partners. Women faced severe physical violence during pregnancy such as having their hair pulled, being slapped, battered, pushed to the floor, pushed down stairs, and kicked in the abdomen. Five major reasons for physical violence were identified; all participants mentioned at least two of these reasons in the individual interviews: 1) had an argument with their husband about issues related to his family members; 2) denied a request to have sex; 3) visited a neighbour’s home “to gossip”; 4) husband came home intoxicated; and 5) only gave birth to daughters.

An 18-year-old, eight months pregnant woman described her experience of IPV:

*"He usually beats me. I don't want to talk about him anymore because I feel irritated when I think of my life. He beats me so much for no reason. He is a shameless drunkard, comes home at midnight drunk and fights with me on unwanted issues. Last night he nearly burnt me - but my father and mother-in-law saved my life (very sad expression)…"*

A 17-year-old girl also eight months pregnant, told her story:

*" I have been being beaten for 3 years (during pregnancies). My husband wants me to give birth to sons; but I am unlucky. I am not able to give him a son, I have only given birth to daughters during the 3 years. I have 3 daughters already and this one is also a daughter (pointing out abdomen). We went for sex identification one month ago and came to know that this one is also a daughter. Since that day, he (husband) started drinking in unlimited amounts. He comes home at midnight and beats me with such a big stick (showing the size with her hand). I don't even remember the number of times he beat me because he beats me till he cools down and blame my maternal home for giving birth to me. I feel so sad and I regret marrying him (tears in her eyes)....."*

Women reported that they had not been physically abused by other family members or neighbours.

#### Psychological Violence During Pregnancy

Twelve women described in detail how they were violated psychologically by their husbands or by other family members. Five women described that scolding, abusive language including accusations of working as a sex worker (“*Randi*”) and of infidelity were some of the typical types of psychological violence that women were exposed to. The most frequently mentioned cause was dispute over sex. Other identified reasons were i) a dispute with her husband’s family members; ii) a conflict with her husband related to his family; iii) her husband complaining of a lack of cleanliness in the house and its surroundings; or iv) her husband coming home drunk.

A 19-year-old, five months pregnant girl, described the psychological violence she experienced:

*" One month ago, I had an argument with my sister-in-law, we nearly had a fight (physically). My husband and mother-in-law separated us and blamed me, which made me feel very bad - especially because my husband did not support me. I was asked to leave the house, which made me really depressed for several days".*

It was clear that women felt extremely vulnerable even though they had not been physically abused. Perceptions of community norms of married women’s behaviour were of no help in the experience of the women. A 19-year-old woman, seven months pregnant, recounted her experience this way:

*"Often I get stressed. Due to this, I just cannot sleep the whole night. I feel like screaming and crying all the time. On those days I regret marrying him. But what to do once a woman is married? She is expected to tolerate all degrees of nonsense acts of her husband and compromise even on the small things. She should not leave him at any cause because he is everything to her and should spend her whole life only with him. If I sit here with him, I get thinner day-by-day because of his misbehaviour towards me. Because of this I feel angry and when I get angry I don't eat food properly for a couple of days. So, whenever we have a big argument I visit my maternal home…"*

The severity of this type of violence was also illustrated in fear. This was especially the case during one interview with a woman who explained that she had not been physically or sexually abused but she was shivering and explained that she constantly felt very nervous.

#### Sexual violence during pregnancy

During the interviews, six young pregnant women described how they had been violated sexually by their husbands. Sexual intercourse during pregnancy is perceived to increase the chances of conceiving a boy child and several women described how the preference of a son was used to coerce her into sexual intercourse. One woman, 24 years old and four months pregnant, described her experiences:

*" Every night we argue a lot over not having sexual intercourse but I have no other alternatives. He himself has studied a lot; he has achieved a master's degree and says that it's good to have sex during pregnancy and if we do so during this period we will give birth to a son. If I say " no it's not, the doctor has told me not to do it" - then he says that if you don't let me have sex, I will go to other girls and marry them. He is my husband after all, so I let him have sex with me…he neither understand me nor my feelings. All he want is to fulfil his desires".*

A 23-year-old woman, four months pregnant, described a similar situation of lack of control of sexual relations with her husband:

*" My husband has kept me in chains (overly protective). He is a very possessive man and controls me by not allowing me to go to others' home, to not talk to men. If I talk to them he will suspect me of having affairs. He does not even allow me to go to my maternal home because I have two unmarried brothers (angry expression). He wants me to have sexual intercourse all 24 h. He forces me for sex, and if I say no then he will say- " Why don't you want to sleep with me? Have you slept with someone else" ? I have to do sex according to his wishes and interests and if I say anything he beats me up" (sad expression).*

Women’s lack of power to withhold their needs made several of the women very upset, yet the concern and fear of their own health, pregnancy and the wellbeing of their current children was described as reasons for giving in to the behaviour or their husband.

#### More than One type of violence during pregnancy

Several respondents were living through more than one type of violence during their current pregnancy. Two women experienced both physical and psychological violence and four were physically, psychologically and sexually violated.

A 23-year-old woman, eight months pregnant, described her experience of psychological and physical violence:

*" Normally the abuse starts when my husband is drunk. He becomes very aggressive and destroys the things that are around him. Just last night he broke the television. He came home drunk and started shouting at me without any reason. He then hit me with a log (showing the size with hand). It was very painful and I was helpless and became very depressed".*

A 24-year-old woman, seven months pregnant, recounted her very recent experience of psychological, physical and sexual violence:

*" I have been sleeping with my daughter in the same bed for two months because my sister-in-law had come and we had only one room to sleep. But she has gone now. Last night, I was sleeping, my husband came home drunk at midnight. He asked me to sleep with him, but I denied. He scolded me badly and forced me to have sex with him but I told him we should not do it because I am seven months pregnant. He then kicked me on my abdomen and slapped me on my cheeks. I also punched him and pushed him away. Then he pulled my hair and pushed me down to stairs and left. Me, holding my 4-year-old daughter, cried the whole night blaming my fate (sad expression)".*

### Coping strategies during violence

Women’s coping strategies when faced with violence were limited. Some women described how they screamed and cried and tried to fight back while a few women mentioned they tried to leave the house and go to an open ground outside the house. But the most frequently mentioned strategy was to tolerate the violent acts of husbands. A 17-year-old girl, 5 months pregnant recounted the following:

*"....I do nothing, just tolerate whatever he does to me because I am the main source of creation of the problem. Being married inter-caste, the doors of my maternal home is closed for me and father-in-law and mother-in-law have not accepted me yet. So I am all alone. I don't have any place to go and express my feelings and he (husband) is everything I have so, I tolerate him and I don't have any other alternatives (tears in her eyes)".*

In total nine women stated that they never tried to stop these acts by their husbands. They said they did not tell anyone or make a single noise while being violated due to many fears and concerns that related to fundamental parts of their life:

i) that others would know about it;
ii) their husband would be shunned in the community and the whole family would get a bad reputation;
iii) to protect their children and the responsibility for taking care of them that women have;
iv) fear that their husbands would leave them;
v) feeling it was mostly their own mistakes;
vi) financial dependency since the husband is the bread winner in the family.

Only six women explained how they actively were seeking support from relatives during violent actions in her pregnancy. Some women shared their problems with their father and mother or went to visit her parents during violent episodes. A 19-year-old woman, seven months pregnant describes:

"*…I share my problems with my mother and father of my maternal home because they are educated and they better understand my problems and support me. I feel shameful to tell others as they might backbite us because we live in the same community".*

Sisters-in-law were the only kin on the side of the husband that women approached for help in the local area they lived. However, these women still expressed a wish for some type of social support that could help them decrease the IPV they were experiencing.

### Women’s views on stopping IPV

Women were asked about ways to reduce, if not end, IPV in urban slums and gave multiple suggestions. A few women pointed to the adverse effects of advanced technology for identifying foetal gender and suggested it should be banned. In addition a few women expressed that women should accept the IPV due to the pride ascribed to men in the local context.

18 of 20 women reported that education for girls and women is the main factor that could strengthen a process of reducing IPV. In addition to education, some other ideas that women gave for stopping IPV were for women to be alert, strong from their inner core of hearts (high self-esteem), and economically independent. A 20-year-old woman, seven months pregnant, explained:

"Women should be independent and strong from the inner core of hearts, should tackle every obstacle that comes on the way and should not be emotionally melted when her husband blackmails her, should take her own decision whether it is right or wrong".

Other woman similarly expressed that the change has to come from women themselves to make IPV visible in the society. A 20-years-old woman, nine months pregnant says:

*"......The major thing to stop the violence is to change the view of society as seeing violence against women as a private matter and that women should maintain their privacy. But it is not so, it is a human rights violation".*

## Discussion

In this study, having a husband who has alcohol use disorder, identification of foetal gender, and refusal to have sex were fuelling factors that instigated IPV among young pregnant women in urban slums. These findings corroborate those in studies from various countries (Chile, Egypt, India, Philippines and USA) in showing a strong link between alcohol use and intimate partner violence [34, 35]. Home-brewing and trading of it is widely acceptable in Nepalese society despite of production and selling of homemade alcohol being illegal. Alcohol is culturally acceptable and used in most of the religious occasions of different ethnic groups, especially *Matawali* (Alcohol-drinking castes such as Tharu and Tamang) [36]. Over the years the use of alcohol is also highly prevalent among other so-called higher ruling castes (Brahmin/*Kshatriya*). Alcohol use is linked to cultural and different social occasions resulting in high social tolerance for the alcohol dependence among male in the Nepalese society. Male with poor economic status usually buy cheap home-made alcohol from the Bhatti (Nepalese speakeasy - a place where homemade alcohol drinks are served illegally). A study conducted in Nepal among men aged 15–60 years found a high prevalence of alcohol dependency (26  %) [37]. It has been indicated that treatment for alcohol dependency is effective in reducing intimate partner violence [38], and such efforts could likely be effective also in the urban slums of Nepal.

We found that easy availability of sex selection technologies is increasing the risk of IPV in urban slums. Other studies have shown that the identification of foetal gender leads to female infanticide in different Asian countries [23]. Our study findings suggest that identification of foetal gender did not led to female infanticide due to poor economic status, but did lead to violence against young pregnant women at the prenatal stage. Young pregnant women from our study recommended banning the identification of foetal gender as a measure to prevent IPV. It is illegal to conduct foetal gender identification and sex selective abortion in Nepal [39]. But it is legal to obtain abortion up to 18 weeks in the case of rape or incest, if the pregnant women’s life is in danger due to pregnancy related complications, or the foetus has congenital anomalies. Other study findings conducted in Nepal suggest that health providers find it difficult to confirm when sex selection results in abortion. However, the widespread availability of technology to identify foetal gender and users’ strong preference for sons is associated with foetal gender identification and sex-selective abortions [39]. The provision of ultrasonography services is relatively cheap (NPR 500-1000/US$ 5–10).

Our finding that refusal to have sex during pregnancy led to violence is also consistent with other study findings conducted in Nicaragua and Zimbabwe [40, 41]. Additional factors such as the influence of alcohol and partner failure to understand pregnancy and emotional changes are known to exacerbate the risk of IPV [35, 41].

Most of the young pregnant women who were victims of violence in this study were trapped in an abusive relationship due to their economic dependence. Other studies suggest that the economic dependency and violence act in a dose–response relationship — the higher a woman’s degree of economic dependency, the more violence she experiences from her partner [42, 43]. Monthly family income of our study participants was very low and some of the study participants were getting financial support from their maternal home to compensate their day to day expenses. This severely affects their ability to acquire food and take care of their children. Food insecurity is an important risk factor not only for the health risk of pregnant mother and their child (malnutrition) but also increased their risk of intimate partner violence. Growing evidence suggests that an association between food insecurity and intimate partner violence [44]. Poverty directly affects the ability to acquire food among vulnerable populations and financial stress increased the risk of IPV. A recent study conducted among US women aged 18 years and older found that women experiencing food insecurity are at higher odds of experiencing IPV than those women who reported being food secure, within the last 12 months [44]. A study conducted in rural Bangladesh also found that women living in households with lower food security status are more likely to experience physical and psychological abuse from their partner [45]. Future studies should investigate the associations between poor economic status linked to food insecurity and intimate partner violence among vulnerable populations in Nepal.

In our study very few participants mentioned coping strategies during IPV (tolerating the husbands’ acts, screaming, crying, going to open ground or fighting back). Some study findings suggest that inadequate coping strategies among women could be correlated with a higher prevalence of IPV victimisation [46]. Studies also suggest that women victim of IPV use maladaptive coping strategies such as alcohol and illicit drug use [47].

In addition to being economically dependent, lack of trust and feelings of shame meant violence survivors in our study were more likely to seek informal care and support from close family members, rather than seeking formal support from “outsiders”. In our study young women did not seek care and support from formal support organisations such as the police because of mistrust. As such they preferred to keep the experiences of IPV private. Lack of other alternatives might also have discouraged young pregnant women from seeking formal support. A study in Northern India on perceptions regarding options available for victims of physical IPV, found that survivors did not seek support from the police or non-governmental organisations because such behaviour is socially inappropriate [48]. The same study also found that physical IPV survivors preferred to use traditional support services (such as jati-panchayat in India) compared to formal organisations. This perspective could be the reason young pregnant women in our study emphasised the need for separate institutions to be established within their community to address IPV. Interestingly, another study found that violence survivors’ disclosure to family members may act as a precursor to formal help seeking [49], and the role of close family members and friends is critical in that regard [50].

### Recommendations to address IPV in urban slums

#### Emphasise the role of health workers to prevent IPV

During regular antenatal care visits of couples, health workers should provide information related to men’s biological role in determining the gender of their children. This effort to help educate and increase awareness in men and their families with strong son preference about the pregnancy process might help to prevent IPV during the prenatal stage. This effort also helps to avoid general misconceptions about pregnancy, such as having sex during pregnancy helps to get a son.

#### Improve coping strategies of IPV victims

Our study findings suggest that women’s coping strategies when faced with violence were limited. Designing and implementing interventions to improve pregnant women emotional abilities to tackle stress might help to prevent mental health consequences of IPV. Evidence also suggests that IPV-survivor women with ability to cope with stress are less likely to suffer from alexithymia and depressive symptoms [46].

#### Involve community people including In-laws in the design and implementation of IPV prevention strategies

IPV survivors in our study seek informal support from relatives and in-laws. They did not seek support from formal support because they believe it is not socially acceptable and feared that it might result in negative consequences. Another study also suggests similar findings [48]. Hence involvement of community people is crucial to design culturally and socially sensitive interventions to prevent IPV among pregnant women.

## Conclusions

Our study shows that the economic and religious-based preference for sons, a woman’s refusal to have sex, and a husband’s heavy use of alcohol are linked to intimate partner violence against women. Our findings also elucidate how easier access to sex selection technologies increases the risk of IPV among young pregnant women. The economic vulnerability of those who have survived IPV is the main reason women tolerate violence, and also acts as a barrier to seeking formal support. Finally, our study showed that interference from close family members helps resolve the risk of physical abuse.

## Additional file

Additional file 1: Interview guide. (DOCX 22 kb)
