# Supplementary material for: Experience of intimate partner violence among young pregnant women in urban slums of Kathmandu Valley, Nepal: a qualitative study
Source: BMC Womens Health. 2016 Mar 5;16:11. doi: 10.1186/s12905-016-0293-7 (PMC4779579; doi:10.1186/s12905-016-0293-7)
Supplement: Additional file 1: — Interview guide. (DOCX 22 kb) [file 12905_2016_293_MOESM1_ESM.docx]

**Additional file 1** Interview guide

**Part I: Socio-demographic Information**

Age: .............

Caste/Ethnicity: .................

Religion: ................

Marital status: ................

Duration of marriage: ...............

Type of marriage: ...................

Age at marriage: ......................

Type of family: ........................

Occupation of:

Respondent: ......................

Husband: ..........................

Education:

Respondent: .....................

Husband: ..........................

Family Income per Month: ...................

**Part II: Questions related to violence**

1. Have you ever been physically violated?
2. If you are physically violated, how is it?
3. What was the physical effect?
4. Ever been psychologically violated?
5. If you are psychological violated, how is it?
6. What was the psychological effect?
7. Ever been sexually violated?
8. If you are sexually violated how is it?
9. What have you done at the moment of violence?
10. How often are you violated?
11. What are the main causes of violence against you?
12. Have you ever been taken for the treatment after violent act?
13. If you have kept the violence on you secret, why?
14. What are the solutions to stop violence against you?

**Physical Violence**

1) During the Pregnancy, have you ever experienced any type of the following acts of violence from an intimate partner, past or present during pregnancy?

1. Slapped you or threw something at you that could hurt you
2. Pushed you or shoved you
3. Twisted your arm or pulled your hair
4. Hit you with a fist or something else that could hurt
5. Kicked, dragged, or beat you up
6. Choked or burnt you
7. Threatened you with, or actually used a gun, knife or other weapon against you

2) During the Pregnancy ,have you experienced any type of the following acts of violence from someone other than an intimate partner, past or current?

1. Slapped you or threw something at you that could hurt you
2. Pushed you or shoved you
3. Twisted your arm or pulled your hair
4. Hit you with a fist or something else that could hurt
5. Kicked, dragged, or beat you up
6. Choked or burnt you
7. Threatened you with, or actually used a gun, knife or other weapon against you

3) During the pregnancy, have you experienced any of the following types of injury as a result of the act of violence by your Intimate partner?

1. Cuts, bruises or aches
2. Eye injuries, sprains, dislocations or burns
3. Deep wounds, broken bones, broken teeth or other serious injuries

4) During the Pregnancy, have you experienced any of the following types of injury as a result of the act of violence by someone other than your Intimate partner?

1. Cuts, bruises or aches
2. Eye injuries, sprains, dislocations or burns
3. Deep wounds, broken bones, broken teeth or other serious injuries

**Psychological Violence**

1. Have you ever been misbehaved by your husband during pregnancy?
2. Have your husband ever threat to expel you from home during pregnancy?
3. Has he ever verbally abused you?

**Sexual Violence**

1. Did your husband ever physically force you to have sexual intercourse with him even when you did not want to?
2. Was there ever a time when you were afraid to say 'no' for sex with your husband?
3. When two people marry or live together,they share both good and bad moments.In your relationship with your husband,do you have such personal experience to share with me?
4. Sometimes husband touch their wive's bodies in a sexual way against their wishes or desire.Have you ever had such experiences? Could you please share such information with me?
5. Did your husband ever threaten you that if you didn't have sex with him he would leave you or go to another women?
6. Did your husband ever force you to do something sexual that you found degrading or humilating?
7. Why do you think that your husband act/behave in such way? Why would your husband force you to perform sexual act? Have you had any other undesired sexual experiences?
8. In your opinion,what are the possible negative effects (consequences) of sexual violence on women lives?
9. Have you ever experienced such problems? What type of problems did you face? When?
10. What did you do to overcome such problems?
11. In your opinion, how can a married women avoid such undesired sexual experiences with their husbands?
12. Have you ever gone to a place or person for help and advice on these matters? When?

If not, Why didn't you go somewhere or contact someone.
